# Transcriptome Changes during the Life Cycle of the Red Sponge, *Mycale phyllophila* (Porifera, Demospongiae, Poecilosclerida)

**DOI:** 10.3390/genes6041023

**Published:** 2015-10-20

**Authors:** Fan Qiu, Shaoxiong Ding, Huilong Ou, Dexiang Wang, Jun Chen, Michael M. Miyamoto

**Affiliations:** 1 State Key Laboratory of Marine Environmental Science, Xiamen University, Xiamen 361101, China; E-Mails: fqiu@ufl.edu (F.Q.); sxding@xmu.edu.cn (S.D.); 2 Department of Biology, University of Florida, Gainesville, FL 32611-8525, USA; E-Mail: miyamoto@ufl.edu; 3 Marine Biodiversity and Global Change Research Center, Xiamen University, Xiamen 361101, China; E-Mails: hlou@xmu.edu.cn (H.O.); dxwang@xmu.edu.cn (D.W.)

**Keywords:** demosponges, ontogeny, gene expression, mRNA, transcriptome profiling, settlement, metamorphosis

## Abstract

Sponges are an ancient metazoan group with broad ecological, evolutionary, and biotechnological importance. As in other marine invertebrates with a biphasic life cycle, the developing sponge undergoes a significant morphological, physiological, and ecological transformation during settlement and metamorphosis. In this study, we compare new transcriptome datasets for three life cycle stages of the red sponge (*Mycale phyllophila*) to test whether gene expression (as in the model poriferan, *Amphimedon queenslandica*) also varies more after settlement and metamorphosis. In contrast to *A. queenslandica*, we find that the transcriptome of *M. phyllophila* changes more during the earlier pre-competent larva/post-larva transition that spans these defining events. We also find that this transition is marked by a greater frequency of significantly up-regulated Gene Ontology terms including those for morphogenesis, differentiation, and development and that the transcriptomes of its pre-competent larvae and adult are distinct. The life cycle transcriptome variation between *M. phyllophila* and *A. queenslandica* may be due to their long separate evolutionary histories and corresponding differences in developmental rates and timing. This study now calls for new transcriptome datasets of *M. phyllophila* and other sponges, which will allow for tests of the generality of our life cycle expression differences and for the greater exploitation of poriferans in both basic and applied research.

## 1. Introduction

Sponges (phylum Porifera) are an ancient group of metazoans that fill an important ecological role in the shallow and deep water communities of both marine and freshwater habitats [1,2,3,4,5,6]. Although their exact phylogenetic location remains in question, sponges originated near the base of the metazoan phylogeny [7,8,9,10], which thereby places them at a key transitional position for comparative analyses on the origins of animal body plans [11], developmental processes and life cycles [12,13], cell/tissue types and multicellularity [14,15,16,17], and gene families and regulatory networks [18,19,20,21]. Sponges are also valued as sources of biological compounds and products with pharmaceutical and other practical benefits to humans [22,23,24,25].

**Figure 1:**
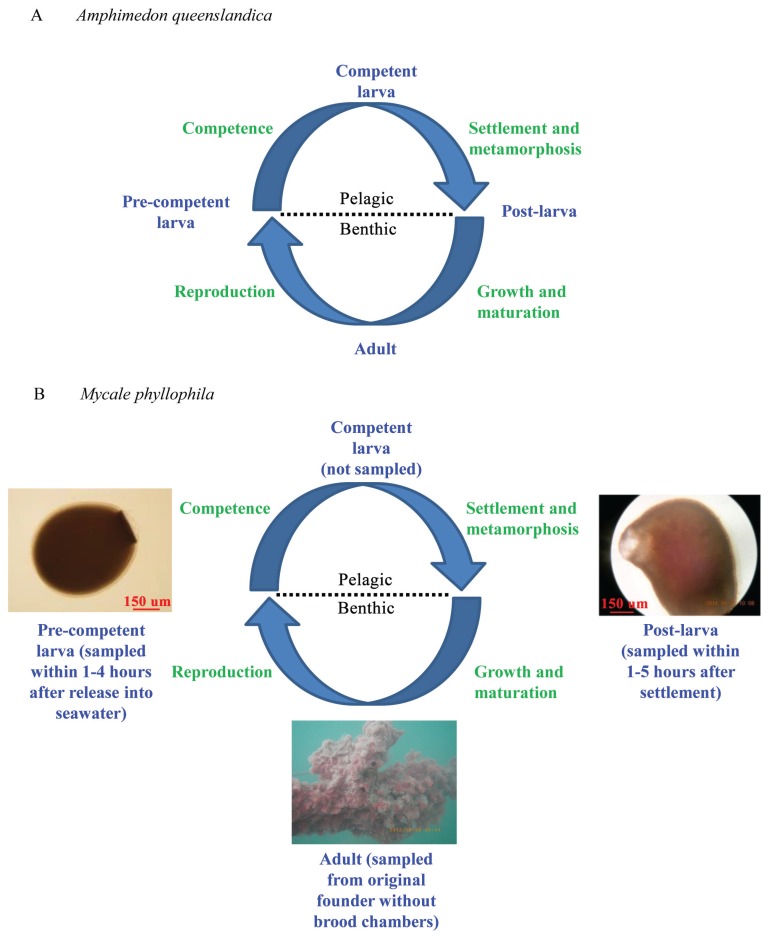
The life cycles of *A. queenslandica* and *M. phyllophila*. (**A**) The life cycle for *A. queenslandica* is from Conaco *et al.* ([30]: Figure 1A); (**B**) Specific sampling times (in parentheses) and photographs are provided for the three represented life cycle stages of *M. phyllophila*.

Because of their broad biological importance to both basic and applied research, numerous studies have sequenced and assembled sponge genomes and transcriptomes [26,27,28,29,30,31,32,33,34,35,36,37,38,39]. *Amphimedon queenslandica* is the first sponge whose genome was fully sequenced and assembled [28] and its transcriptomes from four major life cycle stages (Figure 1A) revealed genome-wide events that occur during its ontogeny [30]. As Porifera constitutes one of the earliest metazoan branches, transcriptome profiling of the sponge life cycle offers an excellent opportunity to reconstruct the first animal program (*i.e.*, “genetic toolkit”) for development and to understand the core traits that underpin multicellularity in animals [8,14,39,40].

Like many other marine benthic invertebrates, sponges also have a typical biphasic life cycle that includes a free-swimming larva and a sessile adult [1,12,41,42]. The switch from free-swimming larva to benthic adult involves the bio-programs of settlement and metamorphosis, during which the developing individual undergoes significant changes in morphology, physiology, and ecology [30,43,44,45,46]. Settlement involves a major shift in the functional and spatial ecology of the developing individual from a pelagic larva to a filter-feeding, sessile adult. Similarly, metamorphosis involves the transformation of that individual’s morphology and physiology, as its undifferentiated cells undergo an extensive reorganization and its larval-specific structures are destroyed while essential juvenile features become completely formed [47,48,49,50].

Conaco *et al.* [30] compared the transcriptomes for the pre-competent larva, competent larva, post-larva, and adult stages of *A. queenslandica* (Figure 1A). In this species of the sponge class Demospongiae [51,52,53], the pre-competent larva stage begins with the release of the embryo from the maternal brood chamber of its parent into the seawater [40,54]. This planktonic stage lasts for a minimum of four hours during which the larva develops the competence to respond to inductive environmental cues for settlement (*i.e.*, the competent larva stage [12,55]). Upon settlement, the competent larva metamorphoses into a benthic juvenile that develops further over the next three days into a tiny sub-adult (*i.e.*, the post-larva stage [46,50]). Thus, as in other sessile marine invertebrates, settlement and metamorphosis occur together during the competent larva/post-larva transition [55]. The post-larva then continues to grow and mature until it produces gametes and is reproductive (*i.e.*, the adult stage).

In this study, we present new transcriptome data and results for three life cycle stages of the red sponge, *Mycale phyllophila* (Figure 1B). This species is also a demosponge [52,53,56], and it is found in the seas of South China, Indo-Malaysia, and Western Australia [57,58,59]. This species has also been reported from the coastline of South Africa [60] and has been recently introduced into Hawaii [61]. Our three life cycle stages for *M. phyllophila* correspond to the pre-competent larva, post-larva, and adult stages of *A. queenslandica* (Figure 1A). Specifically, our post-larvae sample consists of recently settled individuals, whereas that of the adult is from a mature specimen. In turn, the “incompetent” status of our pre-competent larvae sample is based on our unpublished findings that to become competent the recently released pelagic larvae of *M. phyllophila* (as in *A. queenslandica*) must first remain in the seawater for at least four hours.

Conaco *et al.* [30] concluded that the life cycle of *A. queenslandica* depends on many of the same developmental processes and gene groups as used during the ontogenies of other metazoans. In their Figure 4A (which we refer to below as the first gene expression analysis), these authors used Pearson’s *r* as a measure of similarity for quantifying the transcriptome differences between all pairs of their four life cycle stages. Their pairwise *r*s indicated that the transcriptome of *A. queenslandica* changes more after settlement and metamorphosis than during earlier life cycle transitions. Specifically, *r* for their post-larva/adult transition was 0.50, whereas those for their competent larva/post-larva and pre-competent larva/post-larva shifts were 0.76 and 0.75, respectively. Thus, despite the significant morphological, physiological, and ecological changes that underlie settlement and metamorphosis, their *r*s suggest instead that the transcriptome of *A. queenslandica* varies to a greater degree after, rather than during or before, these major processes.

Settlement and metamorphosis are the defining events in the biphasic life cycles of marine invertebrates. In this study, we test whether the transcriptome of *M. phyllophila* also changes to a greater degree during the post-larva/adult transition (as in *A. queenslandica*) or prior to that (as suggested by the significant morphological, physiological, and ecological transformations that underlie settlement and metamorphosis [62,63,64,65]). Our gene expression analyses follow those of Conaco *et al.* [30] to maximize the comparability of our findings with theirs. By providing new transcriptome data and results for another demosponge, our study adds to the growing molecular resources for sponges [14,34,39], which thereby allows for their greater utility in both basic and applied research [6,25,66].

## 2. Experimental Section

### 2.1. Sample Collection and Treatment

Adults of *M. phyllophila* were collected from a natural population in the Dongshan Bay, Fujian Province, China (geographic coordinates 23.797594° N and 117.594821° E) and were then maintained and bred in aquaria at the Department of Marine Biological Sciences and Technology, Xiamen University, Fujian Province, China. Tissue samples of the pre-competent larva and post-larva stages were obtained from the offspring of this breeding laboratory group (Figure 1B). To obtain sufficient RNA for the library constructions, ~1000 and ~500 individuals were separately pooled as the tissue samples for the pre-competent larva and post-larva stages, respectively. To achieve developmental synchronies and minimize individual polymorphisms, each pool consisted of only the progeny for a single gravid adult that hatched within a two hour period. In turn, to avoid contamination with early embryos, the tissue sample of the adult stage was collected from one of the original founders, who was lacking brood chambers at the time of sampling. Specifically, the adult sample was obtained from this founder as a 5 mm biopsy from the apical-to-basal surface of its soma. To avoid RNA degradation, all tissue samples were stored in RNAlater (Ambion, Thermo Fisher Scientific, Waltham, MA, USA) until RNA was extracted [67].

### 2.2. mRNA Isolation and Quality Control

Total RNA was directly extracted from the pre-competent larvae and post-larvae pools with Trizol (Invitrogen, Thermo Fisher Scientific, Waltham, MA, USA) following the manufacturer’s instructions. In turn, total RNA was extracted from the adult sample with Trizol after first washing its biopsy under a stereomicroscope to remove macroscopic debris and then grinding its washed tissue in liquid nitrogen. The mRNA fractions were isolated from the total RNA extracts with the MicroPoly(A) Purist kit (Ambion) following the manufacturer’s protocol. The quality and integrity of the mRNA fractions were confirmed (e.g., by the lack of 18S and 28S rRNA peaks in their electropherograms) with the RNA Pico 6000 chip of the BIOANALYZER 2100 System (Agilent Technologies, Santa Clara, CA, USA) [67].

### 2.3. cDNA Library Preparation and Sequencing

Starting with 250 ng of isolated mRNA, cDNA libraries were prepared for the three life cycle stages with the RNA-Seq Library kit (Gnomegen, San Diego, CA, USA) following the manufacturer’s instructions. The isolated mRNAs were randomly fragmented to generate sequences with an average length of 350 bases. The mRNA fragments were then subjected to first- and second-strand cDNA synthesis and cDNAs of 300–500 bp were size-selected on 1.2% agarose gels prior to their PCR amplifications with primers for paired-end reads (Illumina, Inc., San Diego, CA, USA). A limited number of 15 cycles was used in the PCR amplifications to avoid enrichment artifacts [68]. The amplified cDNAs were sequenced with a read length of 100 bases on a HiSeq 2000 instrument (Illumina, Inc.) at the Berry Genomics Co., Ltd. in Beijing, China.

### 2.4. Sequence Assembly

The raw reads for the three life cycle stages were trimmed with the CLC Genomics Workbench v7.5.1 (Qiagen, Venlo, Netherlands) to remove terminal bases of low quality. Trimmed reads of <20 bases were removed from their datasets as they were too short and ambiguous for use in the sequence assemblies [34]. The quality and quantity of the reads after trimming with different limits was evaluated with FASTQC [69]. These evaluations allowed for the final selection of parameters for maximizing the total amount of high quality trimmed reads with PHRED scores of ≥30 (*i.e.*, with a probability of incorrect base calling of ≤0.001). The trimmed reads for the three life cycle stages were combined to generate a fourth dataset for “all stages”. *De novo* assemblies of the trimmed reads for the four life cycle/all stages were also performed with the CLC Genomics Workbench using the following parameters: Mismatch cost = 2, limit = 8, insertion cost = 3, deletion cost = 3, length fraction = 0.5, similarity = 0.8, minimum distance = 180, and maximum distance = 250.

Contigs of <300 bp were removed from their assemblies as they were too short and ambiguous for use in the sequence annotations and gene expression analyses [34]. For example, Riesgo *et al.* [33] found that very few contigs of <300 bp returned significant BLAST hits for use in their sequence annotations and Gene Ontology (GO) term analyses. Furthermore, even for those with significant BLAST hits, contigs of <300 bp can still be problematic as they are more likely to match different regions of the same gene [70]. Such multiple matching increases the frequency of non-overlapping redundancy, which thereby leads to inflated estimates of gene expression (see below).

### 2.5. Sequence Annotation

The final contigs (*i.e.*, those of ≥300 bp) for the assemblies of the four life cycle/all stages were compared to the entire UNIPROT database [71] with the BLASTX tool of BLAST v2.2.29 [72]. An *e*-value cutoff of 10^−5^ was used in these searches. Contigs with significant BLAST hits were annotated with the GO terms [73] of their top matches with BLAST2GO [74]. The annotated contigs were also checked with the INTERPROSCAN tool [75] of BLAST2GO for the presence of conserved protein-coding sequences for the diagnostic domains of the protein families for their GO terms [37].

### 2.6. Gene Expression Analyses

Conaco *et al.* [30] used multiple technical replicates of the same cDNA library or tissue sample to estimate transcript detection cutoffs for their transcriptome datasets of *A. queenslandica*. Their cutoffs established the degree to which gene expression needed to vary between different life cycle stages to be counted as real and not due to experimental error. On the basis of their technical replicates, Conaco *et al.* concluded that the absence of a transcript in one stage was real and not due to experimental error when it was represented by ≥64 reads in at least one other stage. They further concluded that a ≥4-fold expression difference between stages was also indicative of a real change.

Given our lack of technical replicates and in an effort to maximize comparability, the transcript detection cutoffs of Conaco *et al.* [30], as well as their three gene expression analyses (see their Figure 4), were adopted for our transcriptome comparisons of *M. phyllophila*. Thus, following them, only contigs with ≥64 reads in at least one life cycle stage were counted in our three gene expression analyses. Similarly, our read counts were log_2_-transformed after first increasing them by 1 (the addition of 1 was necessary to avoid taking the log of 0). In turn, our three gene expression analyses were also performed against a reference gene set. In their study, Conaco *et al.* used the genome of *A. queenslandica* [28] as the reference for their read counts and GO enrichment tests. However, in the absence of a sequenced genome for *M. phyllophila*, we used the complete non-redundant contig set for all stages (with “non-redundant” defined according to [70]; see below) as the reference for its transcriptome comparisons [34]. Our gene expression analyses of the read counts were performed with the CLC Genomics Workbench, whereas the GO term evaluations were done with BLAST2GO. Specifically, the following default parameter settings were used in our read mappings: Minimum length fraction = 0.9, minimum similarity fraction = 0.8, and maximum number of hits for a read = 10.

In our first gene expression analysis, the log_2_ (read count + 1)’s for the 8202 contigs of the reference with ≥64 reads in at least one life cycle stage were compared among stages and their pairwise transcriptome differences were quantified with Pearson’s *r* (*cf*. [30]: Figure 4A). Then, in our second gene expression analysis, the log_2_ (read count + 1)’s were compared among stages for the subset of 3827 contigs from above with a ≥4-fold expression difference between one stage and the mean of the other two (*cf*. [30]: Figure 4B). The log_2_ (read count + 1)’s were summarized on a per-gene basis as a heat map for the three life cycle stages. Their pairwise stage differences were then quantified with *r* and with a dendrogram that clustered their rescaled (1 − *r*) distances with the unweighted pair-group method with arithmetic means (UPGMA) [76]. In these first and second gene expression analyses, 95% confidence intervals (CIs) were calculated for the pairwise *r*s with Fisher’s z'-transformation [77].

In our third and final gene expression analysis, the relative frequencies of GO terms for the two life cycle transitions were compared to the reference distribution for the annotated contigs of all stages (*cf*. [30]: Figure 4C). Specifically, our third gene expression analysis included the 1025 and 716 annotated contigs of the reference with a ≥4-fold expression change during the pre-competent larva/post-larva and post-larva/adult transitions, respectively. Enrichment tests [78] were then performed for the two life cycle transitions with the Fisher’s exact test of BLAST2GO. These enrichment tests identified GO terms with gene groups that were either significantly up- or down-regulated between successive life cycle stages. The significance level for these tests was set to α = 0.05 after correcting for the false discovery rate due to multiple testing with the Benjamini and Hochberg [79] method.

### 2.7. Assessing the Coverage of the Transcriptomes

The coverage of the transcriptomes for the four life cycle/all stages was assessed with random subsampling of the original raw reads [33,70] and the ortholog hit ratio (OHR [80]). In the subsampling assessment, seven random subsets, ranging in size by an increment of 12.5% from 12.5% to 87.5% of the original raw reads, were separately generated for the four life cycle/all stages with the CLC Genomics Workbench. The subsamples of raw reads were trimmed and assembled, and their contigs were matched against the UNIPROT database as previously described for the original datasets. The number of contigs with significant BLAST hits and N50 (*i.e.*, the sequence length whereby 50% of the assembled bases were incorporated into a contig [81]) were plotted for each subsample against the corresponding estimates for its original dataset and these plots were then checked for plateaus that were indicative of broad transcriptome coverage [70].

In the OHR assessment, the degree to which full-length expressed genes were sequenced and assembled was evaluated with the following ratio [80]:

(1) $$OHR\;=\;\frac{Base\;pair\;length\;of\;contig\;with\;a\;significant\;BLAST\;hit}{Base\;pair\;length\;of\;top\;BLAST\;match\;for\;the\;contig}$$

Thus, an OHR = 1 implies that the transcript of *M. phyllophila* has been assembled to its full length [70]. Conversely, an OHR >1 suggests instead that the expressed gene contains an insertion or that its top BLAST match includes a deletion. The OHR was calculated for the contigs of the four life cycle/all stages with a significant BLAST hit by the custom PYTHON script, ortholog_hit_ratio_calculator.py, of Ewen-Campen *et al.* [70].

### 2.8. Assessing the Robustness of the Results for the Three Gene Expression Analyses

The robustness of the results for the three gene expression analyses was assessed by the redundancy of the top BLAST hits and the normalization of the unequal raw read counts for the three life cycle stages. In the first assessment, the frequency of redundancy (*i.e.*, multiple contigs with top BLAST hits to the same gene) was estimated in two different ways. In the first, redundancy was estimated as the frequency of multiple contigs with overlapping and/or non-overlapping BLAST hits to the same or different regions of a single gene, respectively [33]. In the second, redundancy was then calculated with only the subset of multiple non-overlapping contigs [70]. Thus, according to the second definition, multiple contigs with overlapping BLAST hits were not considered redundant, because they remained interpretable as distinct matches to the distinguishing aligned sequences of different non-redundant duplicate genes. Conversely, multiple contigs with non-overlapping BLAST hits to the same sequence were still considered redundant, because they remained interpretable as matches to different regions of a single gene, which thereby left them vulnerable to over-counting in the three gene expression analyses. Redundancy in these broad- and narrow-senses was estimated for the four life cycle/all stages with the custom PERL and PYTHON scripts of Riesgo *et al.* [33] and the transcriptome_blast_summarizer.py of Ewen-Campen *et al.* [70], respectively.

In the second assessment, the unequal raw read numbers for the three life cycle stages were normalized by random subsampling of the two larger datasets for the pre-competent larvae and adult (*cf*. [34]). Specifically, subsets of raw reads of equal size as the smallest post-larvae sample were randomly selected from the two larger pre-competent larvae and post-larvae datasets with the CLC Genomics Workbench. The three normalized datasets were trimmed and assembled as before and their assemblies were then analyzed as previously done in the three gene expression analyses. The results for the three gene expression analyses with the normalized datasets were compared to those for the non-normalized samples as a check on the sensitivity of the latter to their original differences in raw read counts.

### 2.9. Ethics Statement

Permission to collect the original adult sponges from Dongshan Bay was granted by the Zhangzhou Ocean and Fishery Bureau, Zhangzhou, Fujian Province, China. *Mycale phyllophila* is not a regulated invertebrate, and thus, no other specific approvals were needed by this study.

### 2.10. Data Deposit

The mRNA transcriptome datasets for the pre-competent larvae, post-larvae, and adult samples of *M. phyllophila* are available under NCBI BioProject PRJNA269144.

## 3. Results and Discussion

### 3.1. Broad Coverage of the mRNA Transcriptomes for M. phyllophila

This study provides new mRNA transcriptome data for three key life cycle stages of *M. phyllophila*, which span across its defining events of settlement and metamorphosis (Figure 1B). By enriching for the poly (A) RNA fraction, our mRNA sequencing avoids the transcripts of the sponge bacterial symbionts [82,83,84], whose poly A tails are much rarer and shorter [85]. Transcriptome sequencing for the three life cycle stages is at a depth of 40.9–50.6 million raw reads (Table 1). After trimming, the numbers of raw reads for the three life cycle stages are reduced by ~10% as sequences of <20 bp are removed from their datasets. Correspondingly, the read lengths are also reduced from their original set size of 100 bases for Illumina sequencing to means of 78.5–79.2 bases for the final trimmed reads. After assembly, the transcriptomes for the three life cycle stages are represented by 39.4–46.8 thousand contigs with mean lengths of 700–755 bp and N50s of 752–851 bp (Table 2). Furthermore, the transcriptome for all stages (*i.e.*, for the reference assembly of the three gene expression analyses) consists of >76.6 thousand contigs that cover >58.7 million bp of expressed coding DNA.

**Table 1:** Summary statistics for the mRNA reads before and after trimming.

Stage	Number of Raw Reads	Number of Trimmed Reads	Mean Length of Trimmed Reads (Bases)
Pre-competent larva	50,575,622	45,196,156	79.2
Post-larva	40,918,798	36,386,512	78.6
Adult	47,692,930	42,343,572	78.5
All stages	139,287,350	123,926,240	78.8

**Table 2:** Summary statistics for the assemblies of the trimmed reads.

Stage	Number of Contigs	Total bp Assembled into Contigs	Mean Contig Length (bp)	Maximum Contig Length (bp)	N50
Pre-competent larva	46,802	32,997,304	705	16,717	761
Post-larva	46,138	32,311,087	700	11,831	752
Adult	39,448	29,801,286	755	23,991	851
All stages	76,640	58,786,388	715	18,064	791

N50 = contig length at which 50% of all assembled bases are incorporated into a contig.

**Figure 2:**
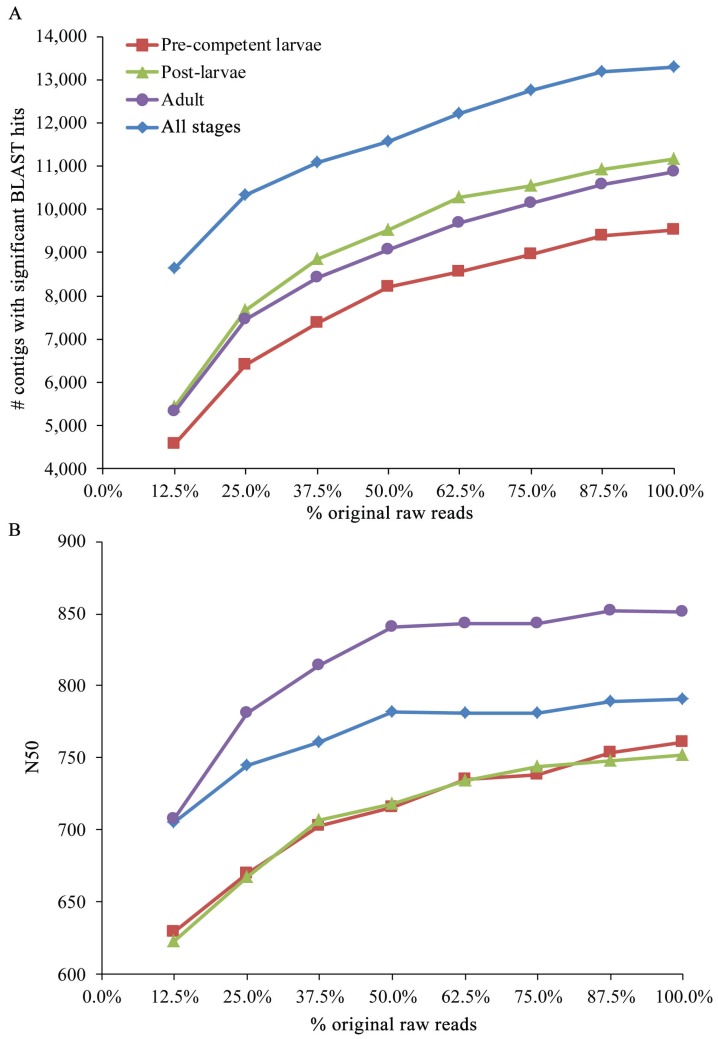
Assessing the coverage of the transcriptomes for the four life cycle/all stages with random subsampling of the original raw reads. (**A**) Changes in the number of contigs with significant BLAST hits as sample size is increased by an increment of 12.5% from 12.5% to 100% of the original raw reads; (**B**) Changes in N50 for these same samples of the original raw reads. In Riesgo *et al.* [33], subsamples of increasing size were generated by adding an increment of 5 million more reads to the previous subset. Thus, our subsamples are more independent than theirs in that each represents its own separate draw of the original raw reads for a stage.

The breadth of the transcriptome coverage is indicated by the subsampling assessment of the original raw reads for the four life cycle/all stages (Figure 2). The numbers of contigs with significant BLAST hits largely plateau by the 87.5% subsamples of the original raw reads for the pre-competent larvae and all stages. Specifically, the assemblies for their 87.5% subsamples include only 1.3% and 0.7% fewer contigs (*i.e.*, 123 and 94 less sequences) than those for their original 100% samples, respectively. Although plateaus are less evident for the post-larvae and adult, the assemblies for their 87.5% subsamples contain only 2.1% and 2.7% fewer contigs (235 and 295 less sequences) than those for their full 100% datasets, respectively. Plateaus are more evident for the N50s of the post-larvae, adult, and all stages as their values begin to flatten by the 75.0%, 50.0%, and 50.0% subsamples, respectively. Although a plateau is less evident for the pre-competent larvae, the N50 for its 87.5% subsample is still only seven bp shorter than that for its full 100% sample. Collectively, the results of the subsampling assessment indicate that additional transcriptome sequencing of the four life cycle/all stages is unlikely to increase the coverage of their expressed genes or their contig lengths to any appreciable degree.

**Figure 3:**
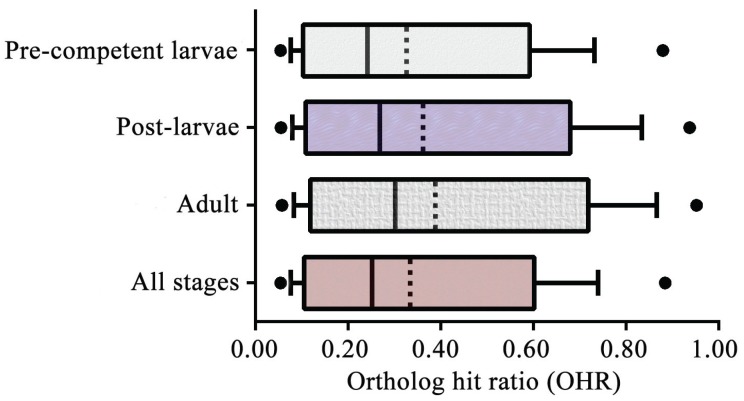
Assessing the coverage of the transcriptomes for the four life cycle/all stages with ortholog hit ratio (OHR). The boxes, error bars, and solid dots demarcate the 25th and 75th, 10th and 90th, and 5th and 95th percentiles, respectively, of the OHRs for all contigs of an assembly with a significant BLAST hit. In turn, the broken and solid lines of each box mark the mean and median OHRs, respectively.

The breadth of the transcriptome coverage is also indicated by the OHR assessment (Figure 3). Mean OHR (± the corrected sample standard deviation) varies from 0.32 ± 0.25 to 0.39 ± 0.28 for the four life cycle/all stages. These OHR means and distributions for *M. phyllophila* are similar to those that have been reported for other sponge and invertebrate species [33,34,70,80,86]. These similarities among different invertebrate species further document that the transcripts of *M. phyllophila* are sequenced and assembled to a sufficient degree for use in the gene expression and GO term analyses.

### 3.2. Transcriptome Changes during the Life Cycle of M. phyllophila

**Figure 4:**
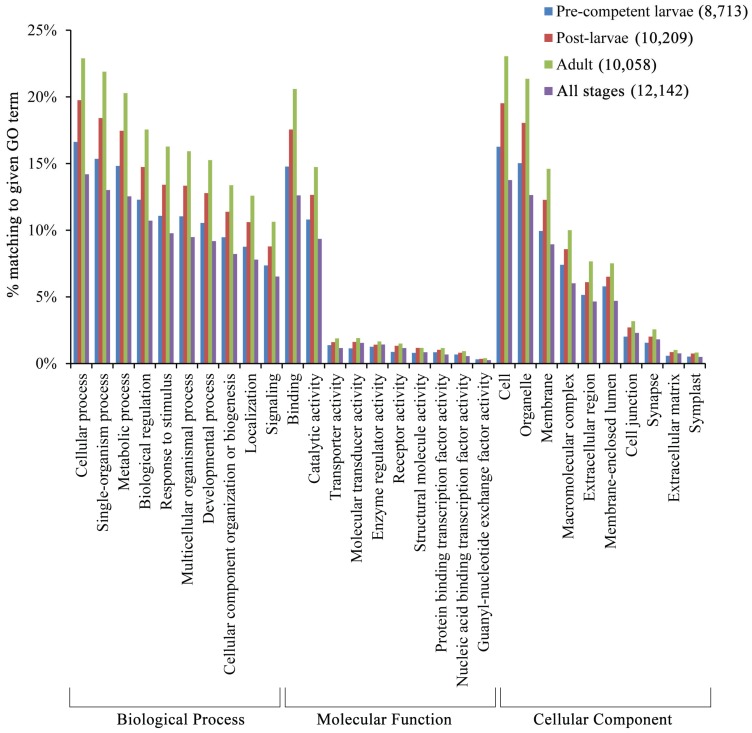
The distributions of Gene Ontology (GO) terms with the highest percentages of top BLAST hits. The percentages of total contigs with a significant BLAST hit to a given GO term are provided for the top ten classes of the four life cycle/all stages under the three highest categories of Biological Process, Molecular Function, and Cellular Component. The numbers of annotated and total contigs are given for the four life cycle/all stages in parentheses and Table 2, respectively.

The top ten GO terms with the highest relative frequencies of significant BLAST hits are summarized for the four life cycle/all stages under the three highest categories of “Biological Process”, “Molecular Function”, and “Cellular Component” (Figure 4). The fourth to tenth top GO terms under Biological Process are for gene groups with direct relevance to a developing individual that is undergoing cell and tissue reorganization, differentiation, and growth. Specifically, these fourth to tenth top GO terms are for development (“Developmental process” and “Cellular component organization or biogenesis”), multicellularity (“Multicellular organismal process”), and regulation (“Biological regulation”, “Response to stimulus”, “Localization”, and “Signaling”). In turn, the top GO term under Molecular Function is for “Binding”. This GO term includes the gene groups for regulatory proteins that control the expression of other genes through their DNA and RNA binding and protein/protein interactions [87,88,89].

**Figure 5:**
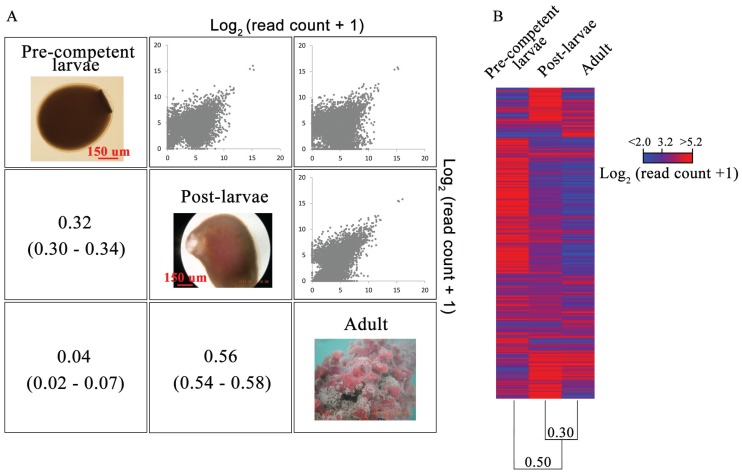
Profiling the life cycle transcriptome changes of *M. phyllophila*. (**A**) Pearson’s *r*s (with CIs in parentheses) and bivariate plots for the log_2_ (read count + 1)’s of the 8202 contigs with ≥64 reads for at least one life cycle stage (below and above the diagonal, respectively); (**B**) Heat map of the log_2_ (read count + 1)’s for the subset of 3827 contigs in Figure 5A with a ≥4-fold expression difference between one stage and the mean of the other two. The pairwise gene expression differences of the heat map are summarized by the UPGMA dendrogram with distance = (1 − *r*).

In the first gene expression analysis, the log_2_ (read count + 1)’s for the 8202 contigs of the reference with ≥64 reads in at least one life cycle stage are compared among the three developmental stages (Figure 5A). Pearson’s *r* for these 8202 contigs is 0.24 less for the pre-competent larva/post-larva transition (0.32) than for the post-larva/adult shift (0.56), and this difference is significant according to their non-overlapping CIs (0.30–0.34 and 0.54–0.58, respectively). Thus, the transcriptomes of the post-larvae and adult are more alike than are those of the pre-competent larvae and post-larvae, which thereby documents that the greater change in gene expression does not occur in *M. phyllophila* after settlement and metamorphosis. In turn, Pearson’s *r* for the transcriptomes of the pre-competent larvae and adult is 0.04, which is still significant for a positive correlation according to its non-overlapping CI (0.02–0.07) with zero (Figure 5A). Correspondingly, a clustering of the data points around the central diagonal of their log_2_ (read count + 1) plot is evident for the transcriptomes of the pre-competent larvae and adult. Still, given that their *r* is close to zero, the transcriptomes of the pre-competent larvae and adult are best interpreted as distinct.

In the second gene expression analysis, the log_2_ (read count + 1)’s for the 8202 contigs of the reference are restricted to the 3827 sequences with a ≥4-fold expression difference between one life cycle stage and the mean of the other two (Figure 5B). Although the focus is now on contigs with stage-specific expression differences [30], the same pattern of transcriptome change is found for the two life cycle transitions. Specifically, the pre-competent larva/post-larva transition is defined by an *r* (0.16) that is 0.24 less than that for the post-larva/adult shift (0.40). Once again, this difference is significant according to their non-overlapping CIs (0.13–0.19 and 0.37–0.43, respectively). Thus, as in the first gene expression analysis, the transcriptome changes to a greater extent during the pre-competent larva/post-larva transition than after settlement and metamorphosis. In turn, the transcriptomes of the pre-competent larvae and adult are once again distinct as they are now defined by a negative *r* of −0.17 (CI of −0.20 to −0.14). This further reduction in *r* for the pre-competent larvae and adult (*i.e.*, from 0.04 to −0.17) is attributable to the use in the second gene expression analysis of only those reference contigs from the first (Figure 5A) with at least a 4-fold expression difference between one stage and the mean of the other two.

**Figure 6:**
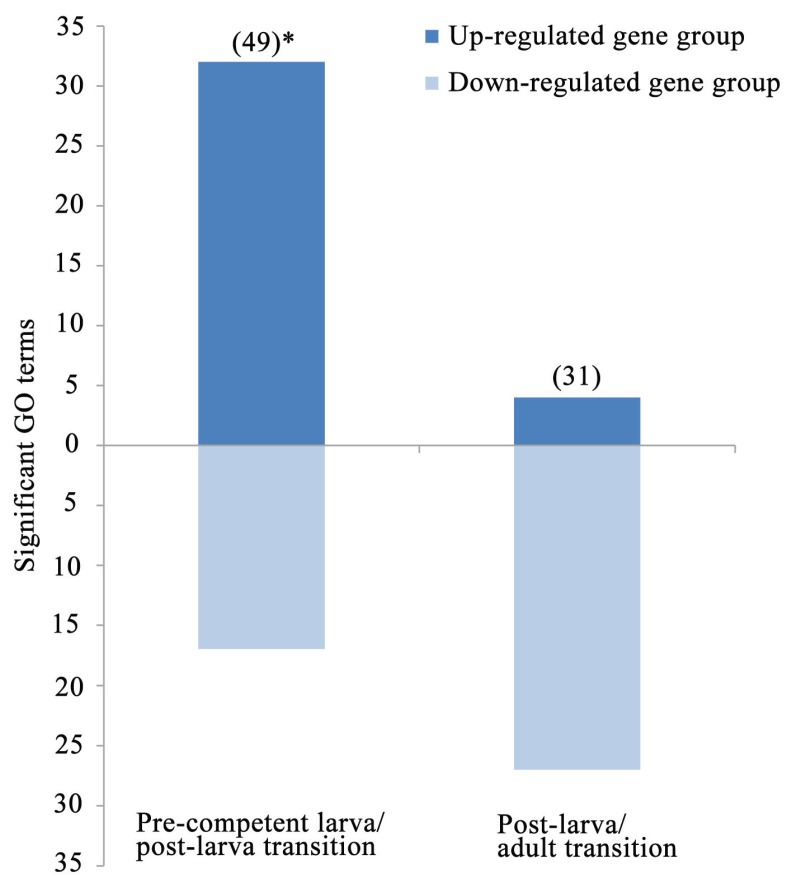
The frequencies of GO terms with significantly up- or down-regulated gene groups during the two life cycle transitions. These frequencies are for the subsets of 1025 and 716 annotated contigs in Figure 5A with a ≥4-fold expression change during the pre-competent larva/post-larva and post-larva/adult transitions, respectively. ( ) = total numbers of significant GO terms (see Table 3 for a list of these designations); * = significant excess of up-regulated classes at *p* < 0.0001.

In the third and final gene expression analysis, the 8202 contigs of the reference are restricted to the 1025 and 716 annotated sequences with a ≥4-fold expression change during the pre-competent larva/post-larva and post-larva/adult transitions, respectively (Table 3). The relative frequencies of the GO terms for the two life cycle transitions are then compared against the underlying reference distribution for all stages. Thus, the focus here is on annotated contigs with transition-specific changes in gene expression [30]. The pre-competent larva/post-larva and post-larva/adult transitions are marked by 49 and 31 GO terms, respectively, with gene groups that are either significantly up- or down-regulated (Figure 6). Importantly, 65% (32) of the 49 significant GO terms for the pre-competent larva/post-larva transition are for up-regulated gene groups. Conversely, the same is true for only 13% (4) of the 31 significant GO terms for the post-larva/adult transition. This difference between the two life cycle transitions in their frequencies of up- and down-regulated GO terms is significant (Fisher’s exact test, two-tailed, *p* < 0.0001). Thus, the pre-competent larva/post-larva transition is defined by an increase in gene expression, possibly because of the significant cellular, structural, and functional changes that occur during settlement and metamorphosis [12,44,62,65]. This early increase is followed by a post-settlement/post-metamorphic decrease in gene expression, most likely because of the greater dependency of the post-larva/adult transition on growth and reproduction rather than on structural and functional transformation [45,46,50].

All eight of the significant GO terms with “morphogenesis”, “differentiation”, and “development” are for gene groups that are up-regulated during the pre-competent larva/post-larva transition (Table 3). These eight GO terms include “Cell morphogenesis”, “Epithelial cell differentiation”, and “Regulation of anatomical structure morphogenesis”. In particular, the up-regulation of “Epithelial cell differentiation” is noteworthy because it supports the conclusion that sponges have true epithelia [16,31,90]. The five other related GO terms with significant up-regulation during the pre-competent larva/post-larva transition are for the morphogenesis and development of structures that are found in the eumetazoans but not in the sponges (e.g., “Branching morphogenesis of an epithelial tube” is for the formation of networks and ducts such as in the trachea and salivary gland of *Drosophila* and the lung and kidney of mammals [91,92]). Collectively, these eight up-regulated GO terms with morphogenesis, differentiation, and development point to metamorphosis once again as a major reason for the increased gene expression during the pre-competent larva/post-larva transition [62,65].

The four GO terms with significant up-regulation during the post-larva/adult transition are for “Hydrolase activity”, “Peptidase regulator activity”, “Peroxidase activity”, and “Phenanthrene-epoxide hydrolase activity” (Table 3). Thus, these four GO terms refer to the three large and diverse enzyme classes/subclass of hydrolases, peptidases, and peroxidases and to the specific hydrolase subgroup of phenanthrene-epoxide hydrolases [93,94,95]. “Peroxidase activity” is also significantly up-regulated during the pre-competent larva/post-larva transition, which thereby documents that the importance of peroxidases continues to increase throughout the life cycle of *M. phyllophila*. The function of phenanthrene-epoxide hydrolases is to detoxify genotoxic polycyclic aromatic hydrocarbons [96,97]. Thus, in addition to playing a role in detoxification [98], other likely biological functions for the four up-regulated GO terms of the post-larva/adult transition include extracellular matrix remodeling and cell adhesion [99], self-recognition, cell-mediated defense, and cytokine-dependent immunity [100,101], and protein degradation in the filter-feeding adult [30].

**Table 3:** Gene ontology terms with significantly up- or down-regulated gene groups during the two life cycle transitions. These significant GO terms are for the subsets of 1025 and 716 annotated contigs in Figure 5A with a ≥4-fold expression change during the pre-competent larva/post-larva and post-larva/adult transitions, respectively. Enrichment tests of the GO terms are performed for the two life cycle transitions with the reference distribution for all stages and with Fisher’s exact test after correcting the significance level (α = 0.05) for the false discovery rate with the Benjamini-Hochberg [79] method.

Pre-Competent Larva/Post-larva Transition		Post-Larva/Adult Transition	
Up-Regulated GO Terms (32)	Down-Regulated GO Terms (17)	Up-Regulated GO Terms (4)	Down-Regulated GO Terms (27)
Actin binding {F:GO:0003779}	Cellular macromolecule biosynthetic process {P:GO:0034645}	Hydrolase activity {F:GO:0016787}	Cell-cell junction {C:GO:0005911}
Axonemal dynein complex {C:GO:0005858}	Cellular protein metabolic process {P:GO:0044267}	Peptidase regulator activity {F:GO:0061134}	Cellular protein metabolic process {P:GO:0044267}
Branching morphogenesis of an epithelial tube {P:GO:0048754}*	Golgi membrane {C:GO:0000139}	Peroxidase activity {F:GO:0004601}	Cytoplasm {C:GO:0005737}
Calcium-dependent protein binding {F:GO:0048306}	Mitochondrial matrix {C:GO:0005759}	Phenanthrene-epoxide hydrolase activity {F:GO:0019118}	Cytoplasmic transport {P:GO:0016482}
Cell morphogenesis {P:GO:0000902}*	mRNA splicing, via spliceosome {P:GO:0000398}		Cytosol {C:GO:0005829}
Cilium movement {P:GO:0003341}	Multi-organism metabolic process {P:GO:0044033}		Endomembrane system organization {P:GO:0010256}
Cilium or flagellum-dependent cell motility {P:GO:0001539}	ncRNA metabolic process {P:GO:0034660}		Establishment of vesicle localization {P:GO:0051650}
Cytoplasmic vesicle part {C:GO:0044433}	Nucleoplasm part {C:GO:0044451}		Exocytosis {P:GO:0006887}
Epithelial cell differentiation {P:GO:0030855}*	Positive regulation of stress-activated MAPK cascade {P:GO:0032874}		Golgi vesicle transport {P:GO:0048193}
Establishment of planar polarity {P:GO:0001736}	Ribonucleoprotein complex {C:GO:0030529}		Homeostatic process {P:GO:0042592}
Extracellular space {C:GO:0005615}	Ribonucleoprotein complex biogenesis {P:GO:0022613}		Macromolecular complex {C:GO:0032991}
Imaginal disc development {P:GO:0007444}*	Ribonucleoprotein complex subunit organization {P:GO:0071826}		Macromolecular complex subunit organization {P:GO:0043933}
Inner ear development {P:GO:0048839}*	RNA binding {F:GO:0003723}		Macromolecule modification {P:GO:0043412}
Lateral ventricle development {P:GO:0021670}*	RNA modification {P:GO:0009451}		Nucleoplasm {C:GO:0005654}
Left/right axis specification {P:GO:0070986}	RNA splicing {P:GO:0008380}		Nucleotide binding {F:GO:0000166}
Locomotion {P:GO:0040011}	Single-organism metabolic process {P:GO:0044710}		Nucleus {C:GO:0005634}
Microtubule motor activity {F:GO:0003777}	Viral life cycle {P:GO:0019058}		Organic substance transport {P:GO:0071702}
Microvillus {C:GO:0005902}			Posttranscriptional regulation of gene expression {P:GO:0010608}
Neurological system process {P:GO:0050877}			Purine ribonucleoside binding {F:GO:0032550}
Neuron projection {C:GO:0043005}			Purine ribonucleoside triphosphate binding {F:GO:0035639}
Neuron projection development {P:GO:0031175}*			Receptor-mediated endocytosis {P:GO:0006898}
Peptidase activity {F:GO:0008233}			RNA binding {F:GO:0003723}
Peroxidase activity {F:GO:0004601}			RNA processing {P:GO:0006396}
Phagocytic vesicle membrane {C:GO:0030670}			Single-organism cellular localization {P:GO:1902580}
Protein heterotetramerization {P:GO:0051290}			Single-organism membrane organization {P:GO:0044802}
Receptor activity {F:GO:0004872}			Single-organism organelle organization {P:GO:1902589}
Regulation of anatomical structure morphogenesis {P:GO:0022603}*			Small molecule catabolic process {P:GO:0044282}
Serine-type peptidase activity {F:GO:0008236}			
Signal transducer activity {F:GO:0004871}			
Sperm principal piece {C:GO:0097228}			
Synaptic cleft {C:GO:0043083}			

( ) = total counts of significant GO terms; { } = GO identification numbers; * = the eight GO terms with “morphogenesis”, “differentiation”, and “development” whose gene groups are significantly up-regulated during the pre-competent larva/post-larva transition (see text).

### 3.3. Robust Results for the Three Gene Expression Analyses

**Figure 7:**
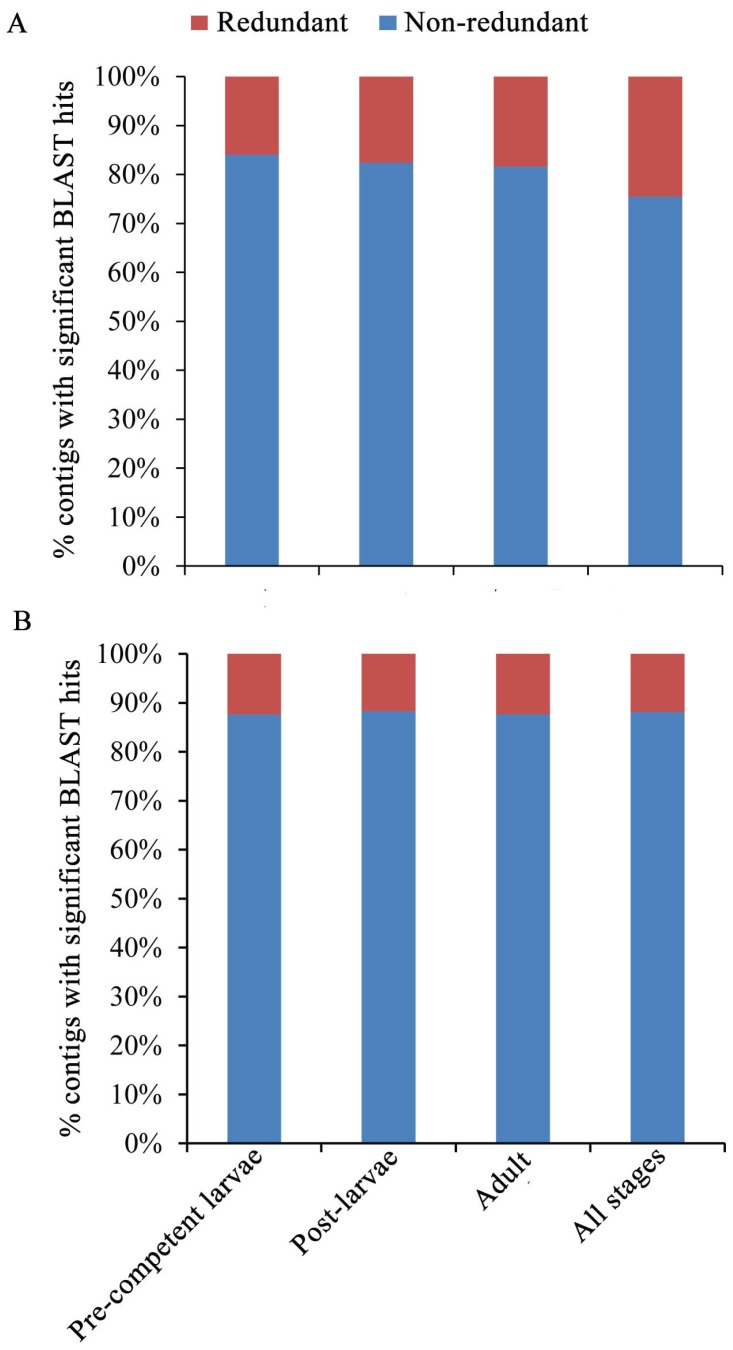
Estimates of redundancy for the four life cycle/all stages. (**A**) Redundancy as broadly defined as the frequency of multiple contigs with overlapping and/or non-overlapping BLAST hits to the same gene; (**B**) Redundancy as narrowly defined with only the subset of multiple non-overlapping contigs. The total numbers of contigs with significant BLAST hits are provided for the four life cycle/all stages in Figure 2.

The robustness of the results for the three gene expression analyses is supported by the two different assessments for redundancy and read normalization. Redundancy can be problematic in that the matching of multiple contigs to the same BLAST hit can lead to over-counting of a gene in a transcriptome analysis [70]. As a check on this potential source of over-counting, redundancy is first estimated as the frequency of multiple contigs with overlapping and/or non-overlapping BLAST hits to the same or different regions of a single gene [33]. Redundancy as measured in this broad sense varies from 15.9% to 24.4% for the four life cycle/all stages (Figure 7A). In turn, redundancy is also calculated such that multiple contigs with overlapping BLAST hits to the same region of a gene are not counted as redundant, but are rather attributed to different non-redundant duplicate genes [70]. Redundancy as measured in this narrow sense is reduced to 11.7%–12.4% for the four life cycle/all stages (Figure 7B). Importantly, our estimates of broad-sense redundancy overlap with those for other sponge and invertebrate species [33,34,70,102], which thereby indicates that the potential for over-counting genes in *M. phyllophila* due to (e.g.,) alternative splicing [39] is at an acceptable level for its gene expression and GO term analyses (Figure 4, Figure 5 and Figure 6 and Table 3).

**Figure 8:**
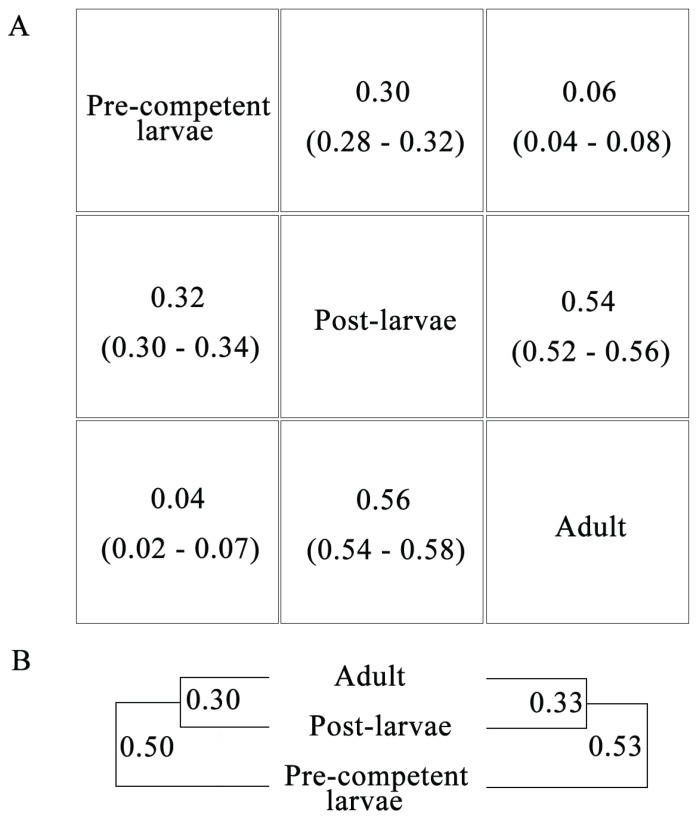
Comparing the results of the first and second gene expression analyses with the original and normalized datasets of raw reads for the three life cycle stages. In the read normalization assessment, the two larger datasets for the pre-competent larvae and adult are reduced in size by random subsampling to equal that of the smallest post-larvae sample (Table 1). The first and second gene expression analyses are then repeated. (**A**) Pearson’s *r*s {with confidence intervals (CIs) in parentheses} for the first gene expression analyses with the original and normalized datasets (below and above the diagonal, respectively; the former correlations are from Figure 5A); (**B**) Dendrograms {*i.e.*, unweighted pair-group method with arithmetic means (UPGMA) with distance = (1 − *r*)} for the second gene expression analyses with the original and normalized datasets (left and right, respectively; the former tree is from Figure 5B).

**Figure 9:**
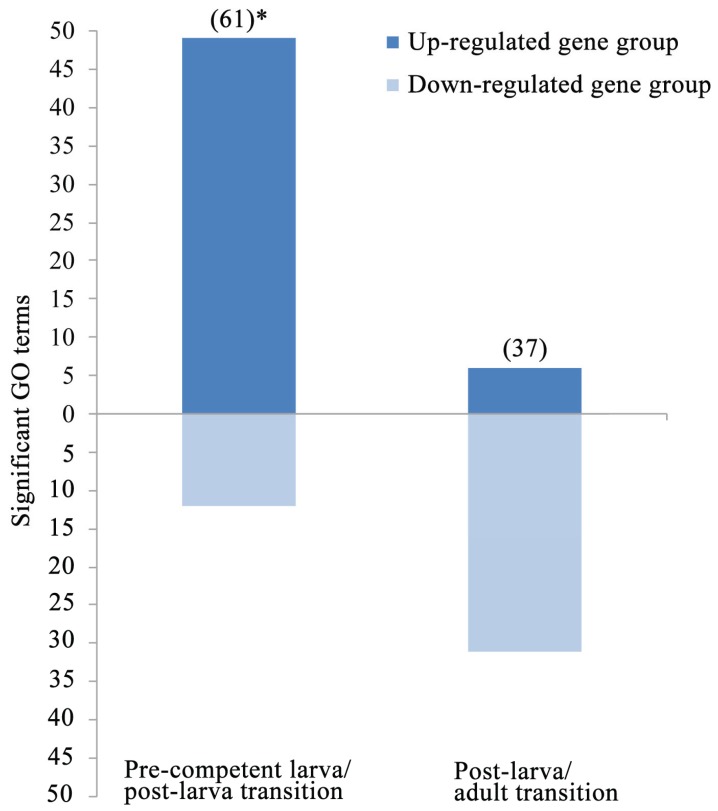
The frequencies of significantly up- or down-regulated GO terms for the two life cycle transitions according to the read normalization assessment of the third gene expression analysis (Figure 6). In this read normalization assessment, the third gene expression analysis is repeated, but with the three equally sized datasets of raw reads for the three life cycle stages. See Figure 6 for other details.

In the second assessment, the two larger datasets of raw reads for the pre-competent larvae and adult are subsampled such that they are now of equal size to each other and to the smallest post-larvae sample (Table 1). The three gene expression analyses are then repeated with these normalized datasets to check on the sensitivity of their results to the original differences in raw read counts. The two larger datasets for the pre-competent larvae and adult contain 9.7 and 6.8 million more reads than the smallest post-larvae sample. Despite these differences in raw read numbers, the Pearson’s *r*s and UPGMA dendrogram for the first and second gene expression analyses with the normalized datasets, respectively, are nearly identical to those for the original life cycle samples (Figure 8). In turn, ~50% more, significantly up-regulated, GO terms are currently recovered for the pre-competent larva/post-larva transition, partly because certain higher-level classes of the original third gene expression analysis are replaced by multiple related groups of the former (e.g., “Actin binding” is now represented by “Actin filament”, “Actin filament binding”, “Actin filament capping”, “Actin filament severing”, and “Actin nucleation”; Table 3). Still, as before (Figure 6), an excess of significantly up-regulated GO terms is found for the pre-competent larva/post-larva transition (Figure 9). Furthermore, as also previously found (Table 3), all nine of the GO terms with “morphogenesis”, “differentiation”, and “development” are restricted to gene groups with significant up-regulation during the pre-competent larva/post-larva transition. Thus, the original main findings of the three gene expression analyses are corroborated by the read normalization assessment, which thereby further documents the robustness of our final conclusions (see below).

### 3.4. The Life Cycle Transcriptome Profiles of M. phyllophila and A. queenslandica are Different

**Figure 10:**
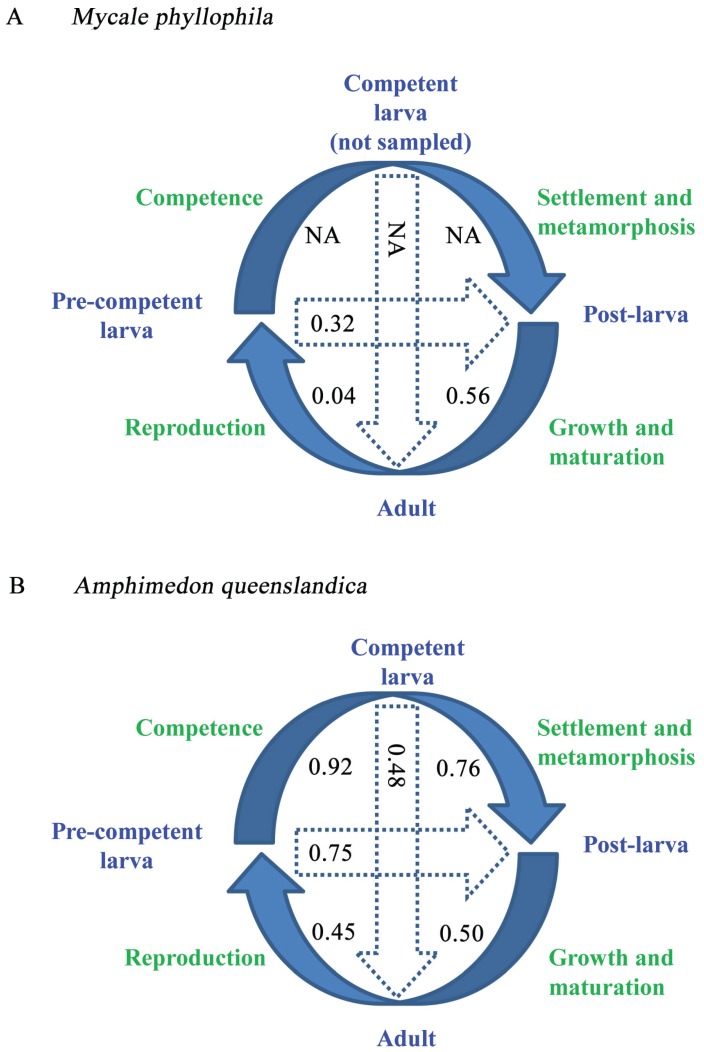
Comparing the life cycle transcriptome profiles of *M. phyllophila* (**A**) and *A. queenslandica* (**B**). Solid and broken arrows connect life cycle stages that are consecutive and non-consecutive, respectively. Pearson’s *r*s for the first gene expression analyses are presented for the two species next to their connections. The *r*s for *M. phyllophila* are from Figure 5A, whereas those for *A. queenslandica* are from Conaco *et al.* ([30]: Figure 4A). (NA) = not available, as a sample of the competent larva stage remains unavailable for *M. phyllophila*.

In the first gene expression analysis, the availability of *r* for the pre-competent larvae/post-larvae comparison, as well as for the competent larva/post-larva transition, of *A. queenslandica* allows for a test of whether the transcriptome of *M. phyllophila* also changes to a greater extent after settlement and metamorphosis rather than during or before these two major events (Figure 10). In *M. phyllophila*, the transcriptome changes to a greater degree during the pre-competent larva/post-larva transition (*r* = 0.32, CI = 0.30–0.34) than after settlement and metamorphosis (*r* = 0.56, CI = 0.54–0.58; *cf*. [62,65]). This trend is opposite of that for *A. queenslandica* where the transcriptome changes more after settlement and metamorphosis than during or before ([30]: Figure 4A). Importantly, this dichotomy holds true regardless of whether or not the pelagic larvae for *A. queenslandica* are competent (*i.e.*, *r* = 0.76 and 0.75, but only 0.50, for the pre-competent larvae/post-larvae, competent larvae/post-larvae, and post-larvae/adult comparisons of this species, respectively).

Still, our lack of a competent larvae sample for *M. phyllophila* compromises our ability to address how much of its transcriptome variation during the pre-competent larva/post-larva transition is due to metamorphosis rather than to competence (Figure 10A). However, in the third gene expression analysis of this species, we find that all eight of the significant GO terms with “morphogenesis”, “differentiation”, and “development” are for up-regulated gene groups during the pre-competent larva/post-larva transition (Table 3). Thus, these eight up-regulated GO terms point to the impact of metamorphosis, rather than of competence, on the changing transcriptome during the pre-competent larva/post-larva transition of *M. phyllophila*.

The life cycle transcriptome profiles of *M. phyllophila* and *A. queenslandica* also differ in terms of their larvae/adult comparisons (Figure 10). In *M. phyllophila*, the transcriptomes of the pre-competent larvae and adult are distinct according to both their first and second gene expression analyses (*r* = 0.04 and −0.17, respectively; Figure 5). Conversely, in *A. queenslandica*, the *r*s for the pre-competent larvae/adult and competent larvae/adult comparisons (0.45 and 0.48, respectively) are both similar to that for the post-larva/adult transition (0.50; [30]: Figure 4A). Thus, in contrast to *A. queenslandica*, the transcriptome of *M. phyllophila* becomes largely reset at the start of every new generation (*i.e.*, during each reproduction from adult to pre-competent larvae). Such a distinct change in the transcriptome has been reported for the polyp/adult transition in the scleractinian coral, *Montastraea faveolata* ([62]: Figure 3), and is consistent with the fact that house-keeping genes (e.g., for “DNA replication”) are also differentially expressed during the life cycle of *A. queenslandica* ([30]: Figure 4C).

### 3.5. A Possible Reason for the Life Cycle Transcriptome Variation between M. phyllophila and A. queenslandica

The life cycle transcriptome profiles of *M. phyllophila* and *A. queenslandica* differ in two important ways: (1) the transcriptome of *M. phyllophila* changes to a greater extent during the pre-competent larva/post-larva transition than after the defining events of settlement and metamorphosis, whereas the reverse is true of *A. queenslandica*; and (2) the transcriptomes of the pre-competent larvae and adult for *M. phyllophila* are distinct, whereas that of the adult for *A. queenslandica* is about equidistant from those of its pre-competent larvae, competent larvae, and post-larvae (Figure 5 and Figure 10; [30]). One potential reason for these life cycle transcriptome differences is that *M. phyllophila* and *A. queenslandica* belong to two distantly related demosponge orders (Poecilosclerida and Haplosclerida, respectively [51,52,53,56]). Poecilosclerida belongs to the “G4” clade of Demospongiae, which is by far the most diverse higher taxon of this class [8]. Conversely, the marine haplosclerids belong to “G3”. The G3 and G4 clades most likely split >650 million years ago in the Cryogenian period [4,103], thereby providing *M. phyllophila* and *A. queenslandica* with considerable time to diverge in their rates and timing of development [50]. Thus, the possibility exists that the early larva of *M. phyllophila* is less advanced in terms of juvenile features than is that of *A. queenslandica* [55]. This possibility is consistent with our finding that, in contrast to *A. queenslandica*, the transcriptome of *M. phyllophila* changes more during the pre-competent larva/post-larva transition than the post-larva/adult shift (Figure 10).

### 3.6. Congruent Findings, Methodological Issues, and Future Directions

Our major findings are corroborated by all of our multiple gene expression analyses and read normalization assessments with different subsets of the full datasets and complements of genes and sequences (Figure 5, Figure 6, Figure 8, Figure 9 and Figure 10 and Table 3). For example, our second gene expression analysis is based on 3827 contigs with stage-specific differences, whereas our third relies on 1025 and 716 annotated sequences with transition-specific changes. Furthermore, our three original datasets differ by upwards of 10 million raw reads, but share equal numbers of sequences after normalization (Table 1). Thus, our main findings are regarded as robust, because they are corroborated by multiple analyses of different datasets that vary in their gene number and content.

Still, our major findings must be viewed with care as our study remains lacking in certain areas. Most importantly, our study is missing biological replicates, which thereby leaves open the possibility that our results are not representative of the underlying developmental processes but are rather biased by an anomalous individual or sample (e.g., an adult with a viral infection or who is temperature stressed). Along these lines, our study is also missing technical replicates, which thereby precludes the experimental determination of our own transcript detection cutoffs. In light of this absence, our investigation adopted the transcript detection cutoffs of Conaco *et al.* [30], both to enhance the comparability of our study with theirs but also because their limits are conservative. However, our adoption of their conservative cutoffs suggests that some genes may have been excluded from our gene expression analyses, which thereby places an even greater premium on our multiple testing for the robustness of our results. Furthermore, rather than correcting for unequal read numbers from the start, our study conducted read normalization assessments after the completion of our other analyses. This strategy allowed us to directly test for the effects of unequal read depths and to assess the robustness of our results with additional “pseudo-replicate” datasets. Still, as read normalization is usually done at the start, additional analyses with a priori normalized data offer another opportunity to evaluate the convergence of our results.

In light of these caveats, our main findings are presented as working hypotheses rather than as final conclusions. As working hypotheses, their purpose is to facilitate the development of novel ideas, to assist in the design of rigorous experiments, and to foster the generation of new comprehensive datasets. Towards these goals, our study now calls for the addition of new transcriptome datasets for the competent larva and other developmental stages of *M. phyllophila* and for the comparable life cycle phases of other sponge species from different genera, orders, and classes. These complementary datasets will allow for tests of many important questions about sponge ontogeny and gene expression. For example, such broad taxonomic sampling will allow for more critical tests of whether the sponge transcriptome changes to a greater extent during settlement and metamorphosis, growth and maturation, or reproduction and what gene groups and regulatory networks are largely responsible for these processes [30,62,65]. In turn, such comparisons will add to the growing molecular resources for sponges [34,39], and thereby, to their expanding utility as key model and reference species in both basic and applied research [6,14,25,66].

## 4. Conclusions

The life cycle transcriptome profile of *M. phyllophila* differs from that of *A. queenslandica* in two important ways. First, the transcriptome of *M. phyllophila* changes more during the earlier pre-competent larva/post-larva transition that spans settlement and metamorphosis, whereas the transcriptome of *A. queenslandica* varies to a greater extent during the later post-larva/adult shift. Second, the transcriptomes of the adult and pre-competent larvae for *M. phyllophila* are distinct, whereas the transcriptome of the adult for *A. queenslandica* is about equally similar to those of the pre-competent larvae, competent larvae, and post-larvae. These differences between the two demosponges may be due to their long separate evolutionary histories and corresponding divergence in developmental rates and timing. Our study now calls for the generation of new transcriptome datasets for *M. phyllophila* and other sponges, which will allow for tests of the generality of our life cycle transcriptome differences and for the greater utility of poriferans as model and reference species in both basic and applied research.
